# Patients’ preferences for antiretroviral therapy service provision: a systematic review

**DOI:** 10.1186/s12962-021-00310-7

**Published:** 2021-08-30

**Authors:** Yihalem Abebe Belay, Mezgebu Yitayal, Asmamaw Atnafu, Fitalew Agimass Taye

**Affiliations:** 1 Department of Public Health, College of Health Sciences, Debre Markos University, Debre Markos, Ethiopia; 2 Department of Health Systems and Policy, Institute of Public Health, College of Medicine and Health Sciences, University of Gondar, Gondar, Ethiopia; 3 Department of Accounting, Finance, and Economics, Griffith University, Brisbane, Australia

**Keywords:** Preferences, Antiretroviral therapy, Mixed-methods, Systematic review

## Abstract

**Background:**

Achieving global targets of adherence to treatment, retention in care, and treatment success remains a challenge. Health system investment to make antiretroviral therapy services more responsive to patients’ needs and values could address these impediments. Appropriate resource allocation to implement differentiated HIV treatment services demands research evidence. This study aimed to provide an overview of the patients’ preferences for antiretroviral therapy service delivery features.

**Methods:**

Electronic databases (PubMed, Web of Science, Embase, and CINAHL) and search engines (Google and Google Scholar) were searched. This review has followed a convergent segregated approach to synthesis and integration. Data from the included studies were systematically extracted, tabulated, and summarised in a narrative review. Studies that analysed preferences for antiretroviral therapy regardless of the method used and published in the English language in any year across the world and HIV positive clients who were 15 years and above on 4th February 2021 were included for this review. The quality of the included studies was assessed using the mixed methods appraisal tool. A thematic synthesis of the data from the findings section of the main body of the qualitative study was undertaken. ATLAS.ti software version 7 was used for qualitative synthesis.

**Results:**

From the 1054 retrieved studies, only 23 studies (16 quantitative, three qualitative, and four mixed-methods) fulfilled the inclusion criteria. The median number of attributes used in all included quantitative studies was 6 (Inter Quartile Range 3). In this review, no study has fulfilled the respective criteria in the methodological quality assessment. In the quantitative synthesis, the majority of participants more valued the outcome, whereas, in the qualitative synthesis, participants preferred more the structure aspect of antiretroviral therapy service. The thematic analysis produced 17 themes, of which ten themes were related to structure, three to process, and four to outcome dimension of Donabedian’s quality of care model. The findings from individual quantitative and qualitative syntheses complement each other.

**Conclusions:**

In this review, participants’ value for antiretroviral therapy service characteristics varied across included studies. Priorities and values of people living with HIV should be incorporated in the policy, practice, research, and development efforts to improve the quality of antiretroviral therapy service hence avoid poor patient outcomes.

**Supplementary Information:**

The online version contains supplementary material available at 10.1186/s12962-021-00310-7.

## Background

Human Immunodeficiency Virus (HIV) infection continues to be a major public health issue throughout the world. Since the start of the pandemic, 75.7 million people have become infected, and 32.7 million people have died from AIDS-related illnesses. Globally by the end of 2019, 38 million people were living with HIV, 1.7 million people were newly infected, and 690,000 people died from AIDS-related illnesses [1, 2]. Over two-thirds (25.7 million) of all people living with HIV reside in Africa [2].

The Sustainable Development Goal (SDG) 3 includes the promise made by the Member States to achieve the end of AIDS by 2030 [3]. To address this issue, the UNAIDS put the target to have 90% of all people living with HIV (PLHIV) will know their HIV status, 90% of those diagnosed with HIV infection will receive a sustained combination of antiretroviral therapy (ART), and 90% of all people receiving ART will have suppressed viral load by 2020. A subsequent 95-95-95 goal is set for 2030 [4]. Moreover, the World Health Organization (WHO) recommends ART for all people diagnosed with HIV (test and treat approach) [5]. Despite such global efforts, ensuring adherence to HIV treatment, retention in care, and treatment success are challenges to low and middle-income countries (LMICs), which require commitment from the patient and the health care team and a productive patient-provider relationship [2].

As the availability of ART for the treatment of HIV/AIDS has increased in resource-limited settings, there has been a move to develop and implement alternative treatment delivery models (also referred to as “differentiated models of service delivery” or DSD) in high HIV prevalence countries to meet the global targets for HIV treatment while maintaining the quality of care [6]. Differentiated ART delivery is a component of DSD. It aims to improve retention and viral suppression by optimizing models of drug and care delivery. Differentiated ART delivery focuses specifically on clients who are on treatment [7].

Differentiated models of ART service delivery typically differ across one or more of the service characteristics (provider, location, frequency, and intensity of care) and aim to provide a more patient-centered service [8, 9]. Four DSD models that focus on stable ART clients are recently identified [10, 11]. They include (1) healthcare worker (HCW) managed groups, (2) facility-based individual models, (3) client managed groups, and (4) out-of-facility individual models. In HCW managed groups, clients receive their ART refills in a group either from a health professional or a lay healthcare staff member. In these models, clients meet in and/or outside of the health care facilities. In facility-based individual models, clients bypass any clinical staff or adherence support and proceed directly to receive their medication. Appointment spacing and the “fast-track” ART refill model are an example of these models. In client-managed group models, clients receive their ART refills in a group in which clients meet outside of health care facilities and manage and run the refills themselves. For out-of-facility individual models, ART refills and, in some cases, clinical consultations are provided to individuals outside of health care facilities; for example, community pharmacies, outreach models, and home delivery [7].

WHO has defined stable individuals as ‘‘those who have received ART for at least 1 year and have no adverse drug reactions that require regular monitoring, have no current illnesses or pregnancy, have not been currently breastfeeding, have a good understanding of lifelong adherence and evidence of treatment success (i.e., two consecutive viral load measurements below 1000 copies/mL). However, in the absence of viral load monitoring and rising CD4 cell counts or CD4 counts > 200 cells/mm^3^, an objective adherence measure can be used to indicate treatment success” [5].

Implementation and strategy prioritization of HIV programs have been difficult in most resource-limited settings [12]. Research and development are required in this regard to bring more innovative ART delivery models. Through the understanding of the aspects of antiretroviral therapy that are of particular importance to PLHIV, it may be possible to develop new models of care that maintain these high levels of adherence, engagement with care, and treatment success. Nowadays, patient preferences studies are increasingly used to inform clinical and policy decision-making in health care in the context of resource constraints [13]. Several quantitative and qualitative studies assessing patients' preferences for ART service have been published, although a little attempt has been made to synthesize the research findings. Previous reviews lacked particular focus and in-depth investigation of ART service provision. Most of the systematic reviews were conducted on the general HIV care aspects (prevention, counseling and testing, service delivery, and ART) [14, 15], included only discrete choice experiment-based studies on HIV treatment service ignoring other designs [16], and focused on HIV care in high-income countries which are not highly affected by the HIV pandemic [14].

This study was a mixed systematic review to contribute to a better and comprehensive understanding of patients’ preferences for ART service provision. It was designed to elaborate on preferences of HIV-positive clients aged 15 years and above, with the goal of aiding policymakers, program managers, and practitioners in Ethiopia and other settings as they expand ART services.

## Methods

### Protocol registration

The Preferred Reporting Items for Systematic Reviews and Meta-analyses (PRISMA) guideline [17] was used to report the result of this mixed-method systematic review. Protocol for this review was registered in the International Prospective Register of Systematic Reviews (PROSPERO) database on ID no: CRD42020212064.

### Databases and search strategy

The literature search was undertaken from inception to 4th February 2021 using PubMed, Web of Science, Embase, and CINAHL databases. In addition, articles were selected using manual search via Google and Google Scholar search engines by combining the search terms used for databases accessed for primary data sources. The SPIDER question framework was employed, and searches used free text and MeSH terms relating to the following: (i) sample (patients); (ii) the phenomena of interest (antiretroviral therapy, antiretroviral treatment, human immunodeficiency virus therapy, HIV treatment, HIV medication, HIV/AIDS therapy, HIV/AIDS treatment, differentiated antiretroviral therapy); (iii) evaluation (preference, patient preference, stated preference, stated choice); and (iv) research type (qualitative, mixed-methods, and quantitative such as conjoint analysis, discrete choice experiment, ranking study, swing weighting study, analytical hierarchy process, best–worst scaling, adaptive conjoint analysis) for all available studies. Besides, the reference lists of included articles were searched manually. The search string was developed using ‘‘AND’’ and ‘‘OR’’ Boolean Operators. The complete search strategy based on keywords is available in Additional file 1.

### Study eligibility and selection

The eligible studies were selected based on the following criteria: (1) analysis of preferences for ART regardless of the method used, (2) being written in English, and (3) sampling of HIV-positive individuals aged 15 years and above. Studies conducted on HIV services other than ART (prevention, counseling and testing, and service delivery); review articles and studies conducted among children, adolescents, pregnant and breastfeeding women and key populations (people who inject drugs, men having sex with men, transgender persons, sex workers, and prisoners) due to special criteria for defining clinically stable clients and key considerations for social and legal issues in accessing ART services were excluded from this review.

All retrieved studies were exported to Endnote version 9 (Thomson Reuters, London) reference manager, and duplicates were carefully removed. Two investigators (YAB and FAT) independently screened thorough review from the title, abstract, and full text of each study. Any disagreements that arose between the reviewers were resolved through discussion.

### Assessment of methodological quality

Two independent reviewers (YAB and MY) assessed the quality of the studies. The Mixed Methods Appraisal Tool (MMAT) [18] was used to evaluate the quality of included studies. This tool includes specific criteria for mixed methods studies, as well as for qualitative and quantitative studies. The tool discourages the use of a scoring system and instead advises to put a detailed presentation of the ratings to provide a better explanation of the quality of the included studies. Any disagreements that arose between the reviewers were resolved through discussion.

Due to the complexities associated with recommendations being derived from both quantitative and qualitative evidence, an assessment of the certainty of the evidence using either the Grading of Recommendations, Assessment, Development and Evaluations (GRADE) or ConQual approach is currently not recommended for JBI Mixed methods research following the segregated approach and not yet assessed in this review [19].

### Data extraction

The data from primary level studies conducted using qualitative, quantitative, and mixed methods were extracted using JBI data extraction tools in the form of customized Microsoft Excel [20]. Two independent reviewers (YAB and AA) extracted the data and cross-checked it to ensure consistency. Discrepancies were solved by discussion and repeating the procedure. The reviewer (YAB) contacted the corresponding author(s) for further information whenever pertinent data was missed from the included studies. Descriptive data were sorted from the studies focused on authors, study aim, year of publication, country, study region, study type, sample size, method of sample recruitment, method of data collection, and data analysis (Additional file 2).

For quantitative studies (and the quantitative component of mixed methods studies), the extracted data included specific details about the method of preference elicitation, attributes (levels), number of attributes, dimension of attributes, and importance of attributes. For qualitative studies (and the qualitative component of mixed methods studies), extracted data included specific details about the themes, key concepts, and relevant quotes appropriate to the review question.

### Data synthesis and integration

This review followed a convergent segregated approach to synthesis and integration, according to the JBI methodology for mixed-methods systematic review [19]. It involved a separate quantitative and qualitative synthesis followed by integration of the resultant quantitative and qualitative evidence. The quantitative data were examined and found to be inappropriate for a meta-analysis due to the occurrence of high heterogeneity in the study designs and results, i.e., different methods to assess preferences, differences in the choice and the definition of attributes and levels, and different ways of reporting results. A thematic synthesis of the qualitative studies was undertaken following the recommendation of Thomas and Harden [21]. ATLAS.ti software version 7 was used for qualitative data synthesis. Both quantitative and qualitative findings were presented in narrative form, including tables and figures. A narrative summary was used for the final integration of the results of the quantitative and qualitative evidence.

In this systematic review, we considered a mixed-methods type of research as studies reported using either one or more qualitative data collection methods (in-depth interviews, focus group discussions, etc.) and one of the stated preference survey methods in the same published study with clear and sufficient reported methods and findings.

In this review, we divided the identified attributes into three dimensions: structure, process, and outcome. These dimensions were based on Donabedian’s model for health care quality and were appropriate to group the wide range of ART service attributes and to have a closer look at what dimensions of ART were most important for the respondents while choosing ART service delivery. The dimension “structure” refers to objective parameters such as material resources, personnel resources and organizational structure. The “Process” dimension includes all activities taking place while giving and receiving ART. The dimension “outcome” denotes the effect of ART service on the health status of patients [22]. Similarly, the impact of each attribute on patient preference regarding ART in each included study was shown by ranking and/or rating the preference (utility) values; and relative importance score, mean, or odds ratio was used depending on the reported data. The relative importance, expressed as a percentage of each of the attributes in influencing treatment decisions, was calculated for each participant by dividing the range of each attribute (utility of highest level minus utility of lowest level) by the sum of the ranges of all attributes, and multiplying it by 100 [23–25]. If a study reported the utility coefficients in a continuous scale of measurement, then the coefficients for discrete levels of each attribute were calculated in reference to a baseline category with the lowest utility value in the same attribute. In the case of the odds ratio reported in a study, the relative impact of each attribute was computed by dividing the highest odds ratio value by the lowest odds ratio value [16]. However, for studies other than discrete choice experiments (rating, ranking, or best–worst scaling studies) included in this review, the reported rankings in the form of mean, relative importance score, or graphical presentation were directly taken.

## Results

### Study inclusion

**Fig. 1:**
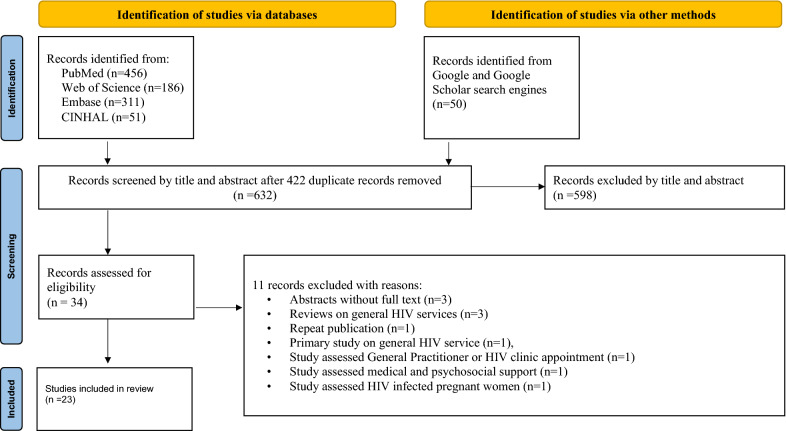
Flow diagram of the included studies for the mixed-methods systematic review of patient preferences for aspects of antiretroviral therapy

The search strategy resulted in 1004 records through (PubMed = 456, Web of Science = 186, Embase = 311 and CINAHL = 51) databases. In addition, 50 studies were accessed manually using Google and Google Scholar search engines. From these, 422 duplicated records were excluded, and from articles screened using their titles and abstracts, 598 were excluded. Therefore, 34 articles were assessed for eligibility. From these, 11 articles were excluded: three were abstracts without full text [26–28], three were review articles focusing on general HIV services [14–16], one study assessed HIV infected pregnant women [29], one study was repeated publication [30], one primary study focused on general HIV service [31], one study assessed General practitioner or HIV clinic appointment [32], and one study assessed medical and psychosocial support [33]. Finally, 23 studies were included in the review. Figure 1 has shown the study selection process.

### Methodological quality of included studies

The methodological quality of included studies varied. All studies presented clear research questions and collected data to address the questions. All the qualitative studies used adequate data collection methods to address the research question, reported the interpretation of results sufficiently substantiated by data, reported the findings adequately derived from the data (for example, the quotes provided to justify the themes were adequate), and provided coherence between qualitative data sources, collection, analysis, and interpretation. About one-fifth of the quantitative studies had samples that accurately represented the target population. Nearly two-thirds of studies had pre-tested questionnaires before data collection. One-fifth of the quantitative studies had reported a non-response rate indicating a low risk of non-response bias. Most quantitative studies used appropriate statistical analysis to answer the research questions. All mixed methods studies reported an adequate rationale for using a mixed-methods design to address the research question. None of the mixed methods had the different components of the study adhere to the quality criteria of each tradition of the methods involved. The integration of both qualitative and quantitative evidence was effective, and results were well interpreted, and there was no divergence of the qualitative and quantitative findings. Overall, no study fulfilled the respective quality criteria. However, in this systematic review, no study was excluded owing to its methodological quality since we were interested in synthesizing all features of ART provision that have been identified as being relevant to PLHIV. The quality assessment matrix is presented in Additional file 3.

### Characteristics of included studies

Our data comprised of seven studies from the African region [34–40], 10 in the Americas [23, 41–49], five in Europe [50–54], and one in both the Americas and Europe (USA and Germany) [55]. Sixteen studies were quantitative [23, 34, 36, 38, 40, 41, 43, 44, 46–48, 51–55] and three were qualitative [35, 39, 42]. Four articles reported both qualitative and quantitative findings [37, 45, 49, 50]. The studies were published between 2002 and 2020. All studies included adult PLHIV in their samples. Twelve studies (eight quantitative and four quantitative parts of mixed methods) recruited the participants using a non-probability sampling technique. Half of the non-qualitative studies were interviewer-administered through paper or tablets. The majority of quantitative and (quantitative part of mixed methods) studies applied mixed logit analytic method whereas thematic analysis was applied in nearly half of qualitative and (qualitative part of mixed methods) studies (Table 1).

**Table 1:** Characteristics of studies included in the review

Author	Aim of study	Publication year	Country	Type of study	Sample	Sampling method	Method of data collection	Method of data analysis
Zanolini [40]	To quantify preferences for a number of potential clinic improvements to enhance retention	2018	Zambia	Quantitative	385 adult HIV patients who were lost to follow-up	Random	Tablet-based interview	Mixed logit
Opuni [36]	To measure preferences for characteristics of hypothetical ART clinics	2010	South Africa	Quantitative	510 adult HIV-infected patients	Not stated clearly	Interview	Random intercept logit model
Tanle [39]	To examine the preferences of PLHIVs concerning ART services	2017	Ghana	Qualitative	145 FGDs and 171 IDIs PLWHIV	Volunteer	FGD and IDI	Thematic analysis
Rabkin [37]	To explore treatment preferences of PLHIV	2020	Zimbabwe	Mixed methods	35 KIIs, 8 FGDs, and 500 survey participants	Purposive sampling of KIIs and convenience sample of FGD and survey participants	KII and FGD, and tablet-based interview	Framework analysis and Fixed effects logit model
Strauss [38]	To assess patient preferences for differentiated HIV treatment delivery model characteristics	2020	Zimbabwe	Quantitative	500 stable adults on ART	Purposive	Tablet-based interview	Mixed-effects logit
Muiruri [35]	To understand preferences for ART packaging	2020	Tanzania	Qualitative	16 adult PLWHIV receiving HIV care	Purposive	In-depth interview	Thematic analysis
Eshun [34]	To determine what DSD features stable patients on ART most prefer	2019	Zambia	Quantitative	486 adult HIV patients on ART	Random	Tablet-based interview	Mixed logit
Mühlbacher [54]	To explore patient preferences regarding treatment of HIV/ AIDS	2013	Germany	Quantitative	218 HIV patients	Not stated	Self-administered online or offline	Random effect logit model
Lloyd [52]	To elicit patient and physician preferences for HIV treatment options	2013	United Kingdom	Quantitative	200 HIV patients	Not stated	Online self-administered	Conditional logit model
Fuster [51]	To determine HIV patients’ ratings of the characteristics of combined antiretroviral treatment	2015	Spain	Quantitative	602 HIV patients attending external consultations in HIV clinics	Casual or incidental non-probabilistic sampling	Self-administered questionnaire	Exploratory factor analysis, cluster analysis, and Student’s t-test for independent samples
Brégigeon [50]	To elicit patient preferences regarding the principal characteristics of ARV therapies and to explore satisfaction of PLWH with their current ARV therapies	2017	France	Mixed methods	Adult patients taking ART:101 PLWH took part in the quantitative and 31 in the qualitative part	Consecutive	Quantitative interview and IDI	Mixed logit and thematic analysis
Moyle[53]	To assess the needs of persons living with HIV regarding ART and to investigate the obstacles currently experienced by European patients when taking or commencing therapy	2003	France, Germany, Italy, Spain, and the UK	Quantitative	504 people with HIV	Posters at HIV specialist care centers and through advertisements in patient-oriented magazines, newspapers, and charities	Interview	Descriptive
Beusterien [41]	To quantify the relative importance of new generation, third-agent HIV drug attributes based on their severity and levels of risk	2005	USA	Quantitative	42 adult HIV-positive individuals	Newspaper advertisement	Computer-administered	Ordinary least squares regression
Miller [45]	To assess patients preferences for different aspects of antiretroviral regimens	2002	USA	Mixed methods	47 FGD among adult HIV patients on ART and 62 patients for quantitative interview	Consecutive	FGD and quantitative interview	Graded pair-preferences method for the quantitative part but not stated for the qualitative part
Eaton [42]	To understand patient preferences for contemporary antiretroviral therapy	2017	USA	Qualitative	28 PLWH > = 19 years old who initiated ART on January 1, 2006	Using flyers, staff referrals, and electronic screens	Nominal group technique	Multi-voting technique
Yelverton [49]	To identify ART characteristics that are important to patients and understand considerations in ART selection from both patient and provider perspectives	2018	USA	Mixed methods	Adult HIV infected individuals on ART: 16 IDIs and 26 for ranking tasks	Patients were recruited by their attending physician, word of mouth, and clinic advertisements	In-depth interviews (IDIs) with HIV patients for the qualitative part and Ranking tasks	Content analysis(qualitative) and count analysis(quantitative)
Sherer [47]	To evaluate the importance of ART attributes	2005	USA	Quantitative	387 adult HIV-positive patients who were currently receiving or had previously received ART	Convenience sampling using flyers	Interview	Paired sample t-tests
Ostermann [46]	To quantify patients’ preferences for key characteristics of modern ART	2020	USA	Quantitative	263 adult PLWH	Flyers and invitation cards; referrals from providers, patients, and members of a community advisory board; and recruitment of patients after clinic appointments	Interview	Mixed logit
Sijstermans [48]	To assess patients’ preferences for HIV treatment in an urban Colombian population	2020	Colombia	Quantitative	224 HIV patients	All patients with HIV in a single City	Self-administered questionnaire	Mixed multinomial logit
Goossens [23]	To elicit patients’ preferences for HIV treatment in the rural population of Colombia	2020	Colombia	Quantitative	148 HIV patients	All patients with HIV in a single City	Self-administered	Mixed logit model
Hendriks [44]	To elicit patients’ preferences for HIV/AIDS treatment characteristics in Colombia	2018	Colombia	Quantitative	283 People living with HIV/AIDS	Not clearly stated	Self-administered questionnaire	Hierarchical Bayes method
Hauber [43]	To estimate the willingness of HIV-positive African American subjects who have chosen not to start ART to accept risks of acute AEs with known outcomes and long-term AEs with uncertain outcomes in exchange for virologic suppression	2009	USA	Quantitative	158 adult HIV-positive but ART-naive individuals	Convenience sampling	Not clearly stated	Mixed-logit
Beusterien [55]	To assess patient preferences for attributes associated with third agent HIV medications	2007	USA and Germany	Quantitative	323 HIV-positive individuals	Advertisements in an electronic newsletter and newspapers and through HIV clinics	Computerized	Ordinary least squares regression

### Quantitative synthesis

#### Method of preference elicitation

Patients’ preferences were elicited with DCE/conjoint analysis method in 12 studies [23, 34, 36–38, 40, 43, 46, 48, 50, 52, 54]; rating exercise [47, 51], ranking exercise [49] and adaptive conjoint analysis [41, 55] in two studies each; and pair-wise comparison [45], and Best–Worst Scaling 1 [44] in one study each (Table 2).

**Table 2:** Methods of preference elicitation

Method of preference elicitation	Number (%)
Discrete choice experiment/conjoint analysis	12 (60%)
Rating exercise	2 (10%)
Ranking exercise	2 (10%)
Adaptive conjoint analysis	2 (10%)
Pair-wise comparison	1 (5%)
Best Worst Scaling 1	1 (5%)

#### Attributes and dimensions

The review showed that the number of attributes ranged from 3 to 26 per study. The median number of attributes used in all included quantitative studies was 6 (Inter Quartile Range 3). Regarding the attributes identified and selected in the included studies, they were clustered into the structure, process, and outcome dimensions of antiretroviral therapy service provision [22]. Overall, the studies used 11 different structure attributes, two process attributes, and ten outcome attributes (Table 3). When summed up, 149 attributes (95 outcome attributes, 47 structure attributes, and seven process attributes) were identified in the included studies (see Table 4). The attributes ‘‘Side effect’’ (n = 13) and ‘‘Efficacy’’ (n = 12) were the two outcome attributes commonly used in the included studies. The most commonly used structure attribute was ‘‘Dosing and administration’’. Other attributes of this dimension that were frequently cited were ‘‘Waiting time at the clinic’’ (n = 5), ‘‘Cost of visit’’ (n = 5), and ‘‘Frequency of visit’’ (n = 4). ‘‘Staff attitude’’ was a commonly used process attribute (n = 4).

**Table 3:** Attributes and dimensions of antiretroviral therapy

Attributes	Number of studies using attribute
Structure attributes (n = 11)	
Dosing and administration	8
Waiting time at the clinic	5
Cost of visit	5
Frequency of visit	4
Distance	3
Operation time	3
Location of service delivery	3
Characteristics related to simplifications	2
HIV clinic branding	1
Buddy system	1
Available clinical evidence or information	1
Process attributes(n = 2)	
Staff attitude	4
Participants/others seen at the same visit	3
Outcome attributes(n = 10)	
Side effect	13
Efficacy	12
Long term health effect	6
Regimen convenience	5
Long duration of drug	2
Interactions	1
Therapy-free intervals possible	1
The drug allows further therapy options	1
It can also be used in case of comorbidities	1
Pregnancy allowed	1

**Table 4:** Overview of attributes, levels, dimension of attributes, attribute importance, and most important attribute

Authors	Attributes (levels)	Dimension of attribute	Attribute importance	Most important attribute
Zanolini	Waiting time at the clinic (1, 3, or 5 h)	Structure	4 (5.20%)	ART supply is given at each refill
	Distance from residence to the clinic (5, 10, or 20 km)	Structure	3 (6.20%)	
	ART supply is given at each refill (1, 3, or 5 months)	Structure	1 (52.70%)	
	Hours of operation (morning only, morning and afternoon, or morning and Saturday)	Structure	5 (3.10%)	
	Staff attitude (rude or nice)	Process	2 (32.80)	
Beusterien_a_	Moderate to severe diarrhea (involving five or more loose stools per day (1%,8% or 16% chance)	Outcome	5 (7.10%)	Chance of developing resistance
	Moderate to severe nausea(5%, 10% or 14% chance)	Outcome	6 (6.90%)	
	Moderate to severe vomiting(2%, 5% or 7% chance)	Outcome	10 (4.70%)	
	Moderate to severe rash(1%, 5% or 10% chance)	Outcome	9 (5.00%)	
	Moderate to severe jaundice(< 1% or 6% chance)	Outcome	10 (4.70%)	
	Moderate to severe dizziness(< 1%, 3% or 6% chance)	Outcome	7 (5.80%)	
	Moderate to severe depression(< 1% or 5% chance)	Outcome	8 (5.50%)	
	Moderate to severe sleep problems(< 1%, 10% or 25% chance)	Outcome	3 (8.60%)	
	Virologic failure(7%, 15% or 21% chance)	Outcome	4 (8.20%)	
	Increasing cholesterol( very low, moderate, or high chance)	Outcome	5 (7.10%)	
	Chance of developing resistance(very low, low, moderate, high, or very high chance)	Outcome	1 (10.30%)	
	Regimen convenience(Fosamprenavir,Fosamprenavir/ritonavir, Efavirenz, Atazanavir, Nelfinavir,Lopinavir/ritonavir)	Outcome	2 (8.70%)	
Opuni	Monthly ART price(12$, 99$, 149$,199$, or 298$)	Structure	3 (23.50%)	Clinic waiting times
	Clinic waiting times(30 min, 2 h, or 5 h)	Structure	1 (33.20%)	
	HIV clinic branding(not branded as HIV clinic in any way, discretely branded as HIV clinic or clearly branded as HIV clinic)	Structure	4 (17.30%)	
	Clinic staff attitudes(kind, respectful, sympathetic, indifferent—neither kind nor rude or rude, disrespectful, unsympathetic)	Process	2 (26.00%)	
Miller	Adverse drug side effects	Outcome	N/A	N/A
	Pill burden	Structure		
	Medication inconvenience	Outcome		
	Regimen potency	Outcome		
Mühlbacher	Life expectancy(maximal or moderate increase)	Outcome	4 (10.02%)	Emotional quality of life
	Long term side effects: improbable (< 20% of patients) or possible (≥ 20% of patients)	Outcome	6 (5.56%)	
	Flexibility of dosing: max. 3 tablets/day or ≥ 4 tablets/day	Structure	5 (6.19%)	
	Physical quality of life: diarrhea or nausea less frequent or diarrhea or nausea more frequent	Outcome	2 (21.97%)	
	Emotional quality of life: disease not obvious for others or disease obvious for others	Outcome	1 (40.71%)	
	Social quality of life: participation in social life possible or participation in social life restricted	Outcome	3 (15.55%)	
Beusterien _b_	Medication resistance	Outcome	N/A	N/A
	Lipodystrophy	Outcome		
	Regimen convenience	Outcome		
	Moderate to severe rash	Outcome		
	Moderate to severe nausea	Outcome		
	Moderate to severe diarrhea	Outcome		
	Moderate to severe sleep disturbances	Outcome		
	Drug failure	Outcome		
	Moderate to severe vomiting	Outcome		
	Cholesterol elevation	Outcome		
	Moderate to severe jaundice	Outcome		
	Moderate to severe depression	Outcome		
	Moderate to severe dizziness	Outcome		
Lloyd	Treatment benefit: 85%, 75%, or 65% chance undetectable viral load at 1 year	Outcome	N/A	N/A
	Risk of rash: Treatment has a 1%, 5%, or 10% risk of rash during the first year	Outcome		
	Risk of kidney stones: In the next five years 0, 10 per 1000, or 37 per 1000 patients will develop kidney stones as a result of this treatment	Outcome		
	Risk of jaundice: Treatment has a 1%, 5%, or 10% risk of jaundice during the first year	Outcome		
	Risk of diarrhea: Treatment has a 5%, 10%, or 17% risk of diarrhea during the first year	Outcome		
	Risk of psychological effects: Treatment has a 10%, 25%, or 50% risk during the first year	Outcome		
	Risk of heart attack: In the next ten years, 0, 6 per 1000, or 40 per 1000 patients will suffer a heart attack as a result of this treatment	Outcome		
	Long term safety profile: Product safety has been established over 10, 5, or 3 years	Outcome		
Rabkin	Location of service delivery: Health facility/clinic close to home or workplace (10 min travel), Health facility/clinic further from home or workplace (45 min travel), Community-based DART services, or At home	Structure	3 (OR:1.70)	Provider attitude
	Participants/others seen at the same visit: Individual or Group	Process	4 (OR:1.30)	
	Type of service provider: Professional health worker who is respectful and understanding, Professional health worker who is not respectful and understanding, Peer/layperson who is respectful and understanding, or Peer/layperson who is not respectful and understanding	Process	1 (OR: 2.40)	
	Times (days and hours) of operation: Workweek only (standard hours: 8 am–4 pm), Workweek with early morning hours (opens at 5 am), Workweek with evening hours (open until 8 pm), or Workweek + weekend hours (7 days a week, 8 am-4 pm)	Structure	7 (OR:1.00)	
	Frequency of routine visits for ART refill: Four times a year (every 3 months) or Two times a year (every 6 months)	Structure	5 (OR:1.09)	
	Total time for a visit, including registration, wait times, and time with providers. It does not include transportation time (30 min, 1 h, 2 h, or 4 h)	Structure	6 (OR:1.05)	
	The total cost of the visit including transportation, direct medical costs (e.g., consultation or booking fee, lab costs if not available at a public facility, non-ARV drug costs), costs of childcare: Free, $1, $3, or $10	Structure	2 (OR:2.36)	
Strauss	Location of service delivery: Health facility/clinic close to home or workplace (10 min travel), Health facility/clinic further from home or workplace (45 min travel), Community-based DART services, or At home	Structure	4 (OR:1.54)	Provider attitude
	Participants/others seen at the same visit: Individual or Group	Process	7 (OR: 0.60)	
	Type of service provider: Professional health worker who is respectful and understanding, Professional health worker who is not respectful and understanding, Peer/layperson who is respectful and understanding, or Peer/layperson who is not respectful and understanding	Process	1 (OR:4.68)	
	Times (days and hours) of operation: Workweek only (standard hours: 8 am–4 pm), Workweek with early morning hours (opens at 5 am), Workweek with evening hours (open until 8 pm), or Workweek + weekend hours (7 days a week, 8 am–4 pm)	Structure	6 (OR:1.10)	
	Frequency of routine visits for ART refill: Four times a year (every 3 months) or Two times a year (every 6 months)	Structure	5 (OR: 1.207)	
	Total time for a visit, including registration, wait times, and time with providers. It does not include transportation time(30 min, 1 h, 2 h, or 4 h)	Structure	3 (OR:1.70)	
	The total cost of the visit including transportation, direct medical costs (e.g., consultation or booking fee, lab costs if not available at a public facility, non-ARV drug costs), costs of childcare: Free, $1, $3, or $10	Structure	2 (OR:1.77)	
Yelverton	ART administration characteristics	Structure	N/A	
	Side effects	Outcome		
	Long-term effects	Outcome		
Sherer	Lowering viral load	Outcome	1 (95%)	Lowering viral load
	Raising CD4	Outcome	2 (94%)	
	Durability	Outcome	2 (94%)	
	Pill burden	Structure	7 (70%)	
	Dosing frequency	Structure	6 (74%)	
	Resistance profile	Outcome	3 (89%)	
	GI SE	Outcome	5 (79%)	
	Appearance SE	Outcome	4 (80%)	
	Cholesterol SE	Outcome	8 (60%)	
Fuster	Dosage	Structure	4 (Mean: 8.41)	Efficacy
	Characteristics related to simplifications	Structure	8 (Mean: 6.40)	
	Diet requirements	Structure	7 (Mean: 7.16%)	
	Tolerance	Outcome	5 (Mean: 8.18)	
	Toxicity	Outcome	2 (Mean:8.70)	
	Interactions	Outcome	6 (Mean:8.13)	
	Efficacy	Outcome	1 (Mean:9.55)	
	Available clinical evidence or information	Structure	3 (Mean:8.64)	
Ostermann	Dosing: Number of pills: one pill once daily, two pills once daily, three pills once daily, one pill twice daily	Structure	3 (17.00%)	Side effect
	Administration: The pills are small, but you must take them with a meal of at least 400 kcal. The pills are large (about 1 inch), but you can take them with or without a meal; or The pills are small, and you can take them with or without a meal	Structure	4 (8.00%	
	Side effects: Moderate diarrhea, -Moderate sleeping problems,-Moderate headaches, Moderate dizziness, Moderate depression or Jaundice	Outcome	1 (44.00%)	
	Long-term effect(over five years): Risk of heart attack, Risk of fracture owing to weakened bones, Risk of new or worse kidney problems, Risk of high cholesterol, or risk of high blood sugar	Outcome	2 (32.00%)	
Sijstermans	Effect on life expectancy: Large positive effects(Live many years more), Moderate positive effects(Live a few more years), or Mild positive effects: Live a short while more (a few months, less than two years)	Outcome	2 (23.00%)	Effect on physical activity
	Effect on physical activity: All physical activities without difficulty, Some physical activities with difficulty, or All physical activities with difficulty	Outcome	1 (25.00%)	
	Risk of moderate side-effects: 1%(Low risk of side-effects), 2.5% (Medium risk of side-effects), or 5% (Higher risk of side-effects)	Outcome	4 (17.30%)	
	Accessibility to the clinic: Less than 2 h, Between 2 and 5 h, or More than 5 h	Structure	3 (20.50%)	
	Economic costs to access controls: Subsidized travel costs, Low travel costs, paid by the patient or High travel costs, paid by the patient	Structure	5 (14.20%)	
Goossens	Effect on life expectancy: Large positive effects(Live many years more), Moderate positive effects(Live a few more years), or Mild positive effects: Live a short while more (a few months, less than two years)	Outcome	2 (26.00%)	Effect on physical activity
	Effect on physical activity: All physical activities without difficulty, Some physical activities with difficulty, or All physical activities with difficulty	Outcome	1 (27.50%)	
	Risk of moderate side-effects: 1%(Low risk of side-effects), 2.5%(Medium risk of side-effects), or 5%(Higher risk of side-effects)	Outcome	4 (16.70%)	
	Accessibility to the clinic: Less than 2 h, Between 2 and 5 h, or More than 5 h	Structure	3 (22.10%)	
	Economic costs to access controls: Subsidized travel costs, Low travel costs, paid by the patient or High travel costs, paid by the patient	Structure	5 (7.60%)	
Eshun	Location of ART pick-up: Clinic or Community	Structure	4 (7.70%)	Frequency of ART pick-up
	Frequency of ART pick-up: Every month or Every 3 months	Structure	1 (62.14%)	
	Time spent in picking up ART:1 h total, 3 h total, or 6 h total	Structure	3 (10.30%)	
	Time spent in seeing the doctor:1 h total,3 h total, or 5 h total	Structure	5 (1.10%	
	Adherence counseling: Individual counseling, Small group counseling (< 6 people), or Large group counseling (> 15 people)	Process	6 (0.65%)	
	Buddy system: Buddy system in place or No buddy system in place	Structure	2 (18.16%)	
Hendriks	The drug has very high efficacy	Outcome	1 (RIS:10.1)	The drug has very high efficacy
	Maximum prolongation of life expectancy	Outcome	2 (RIS: 9.7)	
	Long duration of efficacy	Outcome	3 (RIS: 7.4)	
	The drug improves the physical state	Outcome	4 (RIS: 6.0)	
	The drug does not generate resistance	Outcome	5(RIS: 5.4)	
	Emotional and mental state improved	Outcome	6 (RIS: 5.3)	
	The dosing of the drug may vary	Structure	7 (RIS: 4.9)	
	Once-daily application	Structure	8 (RIS: 4.5)	
	The drug allows further therapy options	Outcome	9 (RIS: 4.4)	
	The drug can be taken along without problems	Outcome	10 (RIS: 3.9)	
	The drug does not affect the appearance	Outcome	11 (RIS: 3.7)	
	Long-term use of the drug is possible	Outcome	12 (RIS: 3.5)	
	It can also be used in case of comorbidities	Outcome	13 (RIS: 3.4)	
	Pregnancy allowed	Outcome	14 (RIS: 3.2)	
	Simple application: only a few tablets	Structure	15 (RIS: 3.1)	
	Long term (hidden) side effects are unlikely	Outcome	16 (RIS: 2.9)	
	The drug does not cause additional costs	Outcome	17 (RIS: 2.6)	
	The drug allows an improved mobility	Outcome	17 (RIS: 2.6)	
	Flexible application	Structure	19 (RIS: 2.2)	
	Social contact opportunities improved	Outcome	20 (RIS: 2.0)	
	Treatment does not require much time	Structure	20 (RIS:2.0)	
	Self-application of the drug is possible	Structure	22 (RIS: 1.8)	
	Therapy-free intervals possible	Outcome	22 (RIS:1.8)	
	Inconspicuous drug intake	Outcome	24 (RIS: 1.7)	
	Rarely occurring diarrhea	Outcome	25 (RIS: 1.2)	
	Rarely occurring nausea	Outcome	26 (RIS:0.9)	
Hauber	The chance that medicine does not work:7%,15% or 21%	Outcome	5	Chance of bone damage
	Chance of having an allergic reaction: None,1%,8% or 12%	Outcome	4	
	Chance of bone damage: None, 1%, 5%, 10%	Outcome	1	
	Chance of kidney damage: None,1%,5% or 10%	Outcome	2	
	What happens if you have bone damage or kidney damage: You don’t know if the problem can be treated successfully, The problem can be treated successfully, or The problem cannot be treated successfully	Outcome	3	
Moyle	Side effects	Outcome	1 (RI:4.1)	Side effects
	Potency	Outcome	2 (RI:4.0)	
	Dosing frequency	Structure	3 (RI:2.6)	
	Total daily pill load	Structure	4 (RI:2.4)	
	Number of pills per dose	Structure	5 (RI:2.1)	

RIS: relative importance score; RI: relative importance; OR: odds ratio

#### Preferences for ART and relative attribute importance

Four studies were excluded from attribute importance analysis due to a study reported mean preference rankings for regimen A and regimen B separately [45], there was a mean percent importance difference between Treatment-Naive and Treatment-Experienced participants [55], attributes were on different underlying scales [52], and the total number of ranks varied due to ties and exclusion of no important characteristics [49] (Table 4). The included 20 quantitative studies were based on a wide range of attributes related to ART service. There was heterogeneity in the results of preference estimates as the attributes were diverse across the included studies. Eight studies evaluated the dosing and administration of drugs. Overall, PLHIV needed a lower pill burden, smaller pill size, and lower frequency of drug-taking [44–47, 49, 53, 54]. The preference value ranking attached to the attribute ‘‘Clinic waiting time’’ varied, ranging from 1 to 6 among five studies [34, 36–38, 40]. In general, participants wanted a shorter duration of waiting time till the upcoming appointment. The participants choice rank for the attribute ‘‘Cost of visit’’ was heterogeneous across the five studies [23, 36–38, 48]. Participants did not want to pay for ART services. The evidence from 4 studies found that participants preferred less frequent clinic visits [34, 37, 38, 40]. Good provider attitude was highly valued by participants ranked either first in two studies [37, 38] and second in another two studies [36, 40]. The better efficacy of antiretrovirals (ARVs) was highly valued by participants, as shown by the 12 studies [23, 41, 43–45, 47, 48, 50–54]. Participants of the included studies also valued reduced or no side effects of ARVs as reported in the 13 included studies [23, 41, 43–49, 51–53, 55]. Similarly, participants had more value on low or no long-term health problems following taking medications as reported in the included six studies [43, 44, 46, 49, 52, 54] (Table 4).

### Qualitative synthesis

Qualitative evidence about patient preferences for ART service provision was reported in seven studies [35, 37, 39, 42, 45, 49, 50]. Data from qualitative studies were also organized into the structure, process, and outcome dimensions of the quality of care [22]. The thematic analysis produced ten themes under structure, three themes under process, and four themes under outcome dimension. However, the themes of inconvenience and novel ART delivery methods were categorized under both the structure and outcome dimensions. Table 5 summarizes initial concepts, emergent themes, final themes, supporting quotes, and dimensions of the final themes.

**Table 5:** Summary of initial concepts, emergent themes, final themes, supporting quotes, and the dimensions of the final themes

Initial concepts	Emergent themes	Supporting quotes and contributing studies	Final themes	Dimension of final themes
Receiving ART information from TV and Radio	Media source	“I got the information on ART from the TV and radio. They said there is a drug that can help AIDS patients to live longer.” [39] “At the hospital: I heard about it from the hospital… After testing when I was positive, the nurse informed me of the availability of ART.” [39] “I heard of the ART drug from the hospital when my husband brought me, and I was tested positive. The doctor said there is a drug that will make us stronger all the time.” [39]	Source of information on ART	Structure
Hearing about ART information from the hospital	Health care providers at the hospital			
Being told by a Nurse about the availability of ART				
Being informed by the doctor about the availability of a drug				
Hospital education and preparation	Health facility service features	‘‘For ART drugs, we prefer the hospitals because these drugs are not just any drugs to be administered at home, because there is a lot of education and preparation before these drugs are given.’’ [39] ‘‘We prefer facility-based because it will be a big problem if it is home-based. Anytime I come here, people see me coming to the hospital for a different purpose other than coming for the ART drug, and it is better that way. But if it is home-based, it will make us think about it, and that alone can kill us early.’’ [39] ‘‘I prefer facility-based like the ***** hospital because when we all come to meet at the center to take our drug, we share our problems together and laugh and make ourselves happy which help take some of our sorrows away.’’ [39] ‘‘I prefer the facility-based service to home-based because there are some people who have the disease but don’t want their relations to know because of stigmatization. Home-based treatment will increase stigmatization.’’ [39] ‘‘...Everyone goes to the clinic, so no one will know why you are coming here [to the clinic], you may say you have a headache…Home delivery is a no....’’ [37] ‘‘... I don’t like them [medications] to be brought home because my neighbors may know, and I may lose hope. I will lose hope forever.’’ [37] ‘‘...getting treated at the clinic is important...because there are times when you will be at the clinic, you can discuss until you are satisfied... than at home or at shops where you can just go and pick your pills there is no time for discussions.’’ [37]	Preferred place for ART services	Structure
Meet and interact with colleagues				
An opportunity for discussion with providers				
Access to psychosocial support				
Avoids stigma and discrimination				
Open location of some ART clinics deter ART access				
Drugs can’t be delivered at home	Home-based service features			
Home-based treatment will increase stigmatization				
Home-based service may demand training more ART provider				
Health workers know how drug improves patient context	Health worker	‘‘I will prefer health workers who have been rendering the service because they know how the drug has been improving our situation.’’ [39] ‘‘We want the doctors and nurses because they have been trained, so they know all about the drugs and the disease.’’ [39] ‘‘PLHIVs should be trained to help in giving out the drugs. If this is done, it will encourage us to feel free to go for the drugs because if a PLHIV gets there and sees the colleague HIV patient giving the drug, they will not feel shy again.’’ [39] ‘‘I think it is best if ‘foreigners’ come because the indigenes would let others know about your status. As of now, those who give the drug to us are not from this community, and they relate to us very well. I like the way they relate to us, so if they continue, it will be better.’’ [39]	Preferred person to deliver ART services	Structure
Doctors and nurses know about drugs and disease due to their training and experience				
PLHIVs should be trained to help in giving out the drugs	Trained PLHIV			
Trained PLHIV encourages the patient to feel free to go for drugs				
Trained PLHIV avoids patients' feeling shy				
Choosing foreigners since indigenes might break confidentiality	Foreigners			
Foreigners relate with patients well				
Nice and respectful	Provider’s attitude	‘‘The health workers behave towards us very well. They handle us like we are siblings. In the beginning, before they put you on these drugs, they ask you to bring a family member along. It is not difficult to get such a person. It should be someone whom you believe will not go and spread the information that you are infected. When they put you on the drugs, you wouldn’t have to come with anybody to the hospital again.’’ [39] ‘‘They are generally good. She will ask which part of your system is disturbing you. They don’t meet us with frown faces; we converse with them nicely.’’ [39] ‘‘We expect that when we come here for a service that we are treated like normal people in the same way that someone with flu or a headache is treated and not to be labeled as ‘the ones who have come for medication’....’’ [37]	Provider’s attitude	Process
Provider request a family member of own choice before putting on ART				
The provider doesn't request a family member when putting on ART				
Nice and sympathetic				
Respect and confidentiality				
Unable to afford the cost of transport	Transportation cost	‘‘The distances are far. Due to this, there are some people who hardly come for the drugs on a regular basis because they cannot afford the cost of transport involved.’’ [39] ‘‘My problem is transportation. Where I stay is far from where I take the drugs. It cost me about GHC5 anytime I come for my drugs. Under such circumstances, if you don’t have somebody to support you financially, it will be difficult for you to collect your drugs regularly as required.’’ [39] ‘‘The drugs should be given to us free of charge. Sometimes, for one year, I will not have money, but they give the drugs to me.’’[39] “My major concern is that I will not be able to get access, or to get it for free more, because of the change of the government.” [42] “Some people, I mean, you have, it’s affordable. Then you have to come to where the insurance will pay so much, and then you have co-payment. And that’s still expensive for some people.” [42] “If I need to go back to Mexico, how will I get it?” [42] ‘‘If I had to pay for the medicine myself, I’d probably be dead. [.] Because I couldn’t afford it.’’ [49]	Cost of ART medications	Structure
Transportation cost				
Out-of-pocket cost	Drug cost			
High insurance co-payment				
The patient recommends a free drug cost				
Patient willing to pay whatever amount required as a drug is available				
Long waiting time	Waiting time	“At times, you spend a lot of time over there, from 8:00 am to 3:00 pm, due to huge numbers coupled with a small number of health workers.’’ [37, 39]	Time spent at clinics in ART pick up	Structure
Shorter waiting time				
Flexible clinic hours	Times of operation	“It depends on your schedule for collecting the ART drugs; yesterday, for instance, by 1:30 pm, everybody had been served, and the center had closed. There are some days when the pressure is quite high, particularly on Fridays but on Wednesdays, there are few people.’’ [39]	Times (hours and days) of operation	Structure
Day of schedule				
Less frequent visit/appointment	Visit frequency	Not applicable [37]	Visit frequency	Structure
Once or twice a year visit with larger supplies of ART dispensed at each visit				
People want to be seen individually	Individual model preference	“I think people want privacy in general. No one wants their health status to be known, so people want to be seen individually, and I think there is no one who put a sticker there.’’ [37] “People are a problem, I want to come alone, but I do not mind coming with a family member so that they know where I get my medication just in case I get sick.’’ [37]	Individual model preference	Process
The patient wants to come alone but do not mind coming with a family member				
Good relationship and open communication	Good relationship and open communication	‘‘I am not my diagnosis. I am somebody. [.] So I think he is starting to understand that, that I need more than ‘‘your levels are fine.’’ He literally turns the screen and scrolls down and shows me the levels and recent tests and what he wants and what he thinks how we should move forward.’’ [49] ‘‘You know, I would talk to my doctor, what’s his experience with the medication and what he knows about the medication [.]. I would pump my doctor for information, what he knows [.], how an adverse reaction relates to the majority of people on this medication as opposed to [a] small group.’’ [49] ‘‘And fortunately that I have excellent communication with my doctor because if I have an issue with home or something comes up, I can email him, and I will hear back from him immediately. So that helps a lot to have good communication.’’ [49] ‘‘Ultimately, it is that patient’s decision, but I take the advice of what my doctor offers.’’ [49] ‘‘Like I said, I did it because the doctor prescribed them for me. [.] And I figured she would know what was best for me. She’s the doctor, and I’m not. [.] So whatever she prescribed, I took. [.] Just as simple as that, you know.’’ [49]	Patient involvement, relationships with providers, and shared decision making	Process
Patient as a final decision maker	Shared decision making			
The patient decides based on the doctor's recommendation				
Paternalism				
Single tablet daily	Concerns of pill burden	‘‘One a day. That’s a lovely thing to have to do.’ ’[49] ‘‘One of the main things I would require is for that medicine to also be like one tablet a day, but the amount of that new med is not as important as what it brings, you know, what it has to offer.’’[49] [Interviewer:] ‘‘Let’s assume [.] you would go to a regimen which means twenty-seven tablets, but there would be not such a high risk for having liver problems, and your renal system would be great, would you do it?’’ [Subject:] ‘‘I would try it, yes.’’ [49] [Interviewer:] ‘‘So there’s a drug which would cause dizziness, but you could go down to one tablet in the morning, and there’s a drug which doesn’t cause dizziness, but you would have to take two tablets two times daily.’’ [Subject:] ‘‘Two tablets two times a day. I probably would go with the two tablets twice a day.’’ [49]	Pill burden and pill size	Structure
The problem of swallowing big pills				
Willingness to accept higher pill burden in exchange for reduced side or long-term effect				
The patient prefers drugs in the form of a liquid, capsule, or injection to either avoid swallowing pills or to lower the intake frequency with an injection once a week or month	Drug administration choice	“The other thing is I would like for or want this medicine to be in a convenient 3, 6, or 12-months injections.” [42] “One 37-year-old female participant raised the possibility of monthly ART injections, stating this would allow them to receive their medication at the clinic and eliminate the risk of unwanted disclosures in their daily lives”[35] ‘‘Some pills have an after taste once you swallow it, and it can be a severe case to the point where you just do not want to take it.’’[49]	Drug administration	Structure
Feeling burdened due to daily drug taking				
Unpleasant drug taste				
Privacy of drugs	Perceived challenges with ART packaging and self-repackaging	“It is easy for people to recognize the ARTs when they are in their boxes. Even a child can recognize them.” [35] “if the medicines are in the bottle, it’s a normal thing to produce some kind of noise, and this is not for ARTs only; it’s for all other types of medicines.” [35] “Some of us stigmatize ourselves, thinking that if people see us carrying the medicines in the box, they will know that we have HIV.” [35] “If people see [the pills], they will not respect the person who takes the medicines because they will know your status.” [35] “Maybe they are afraid to be stigmatized. You know people live in different circumstances, so it depends on the community that the person lives in and how they perceive the problem.” [35] “There are some patients who don’t like to be seen by others; they hide even while at the clinic. They hide because they are afraid to be seen by the people they know, especially if it’s their first time.” [35] “Repackaging can affect the quality of the medicines and the health of the person who takes the medicines because if the medicines have been repacked and are not kept in a proper bottle, that can allow water or air to come into contact with the medicines.” [35] “The health workers advise us not to repack the medicines because, if we do so, we will make the medicines be less efficient.” [35] You know we are human beings, and we are all different. People do different things for different reasons, but we are all adults. We were told the disadvantages of repacking the medicines, and we understand them, but still, people repack. What can be done then? [35] “There are patients who are supposed to take two drugs in a day, morning and evening. So, if you repack, how are you going to remember which ones to take?” [35] There is not any relationship between the way the medicines are packed and taking them. If someone commits to taking the medicines as prescribed, it doesn’t matter how the medicines are packed [35] If the person is repacking the medicines because he is afraid that other people will know that he has HIV, this can affect the way he takes the medicines. Because if, for example, it’s time to take the medicines and there are people present, this person will be afraid to take them because he will be thinking the people he is sitting with will know that he has HIV. In this way, he will not be taking the medicines as prescribed [35] “I think they should remove the box because of its size. If they remove the box, the bottle will not be easily seen, especially for men who like to put the medicines in their trouser pockets.” [35] “When I came here the first time, I saw other people removing the box, and I decided to do the same” [35] “I think the major reason is the size of the bottle. Sometimes when you walk the medicines will make noise, so you will be afraid because you haven’t accepted your [HIV diagnosis]”[35] “I would suggest the medicines be given in blister packs. Blister packs are transparent, but they are well packed. This will make them easy to carry, and patients will feel comfortable putting them in their bag because they do not make noise. "[35]	ART packaging and self-repackaging	Structure
Easily identifiable ART box				
Bulky packaging				
Plastic bags reduce noise				
Perceived stigma				
Appropriate current packaging	Perceived benefits of current ART packaging			
Current packaging valuable				
Health providers advise not repacked ART				
Continued practice of repackaging				
The connection between self- repacking and poor adherence	Relationship between self-repackaging and perceptions of ART adherence			
The link among stigma, repacking, and adherence				
The pillbox should be removed	Recommendations for patient-centered ART packaging			
Boxes should be small in size and non-descriptive				
Learn self-repackaging from others				
Inconvenient box discarding at home				
Disliked pill bottle				
Monthly ART injections				
''Small pouch''				
Blister packs				
Regimen potency	Medication controls HIV	‘‘Want a regimen that was most effective at fighting HIV and prolonging life, regardless of side-effect severity, complexity, inconvenience or pill burden" [45] “Maintain the levels of HIV in control” and “to help me feel better … even on bad days” [42] ‘‘Well, first, of course, would be the efficacy of the medication, what its track record is and everything.’’[49]	Efficacy	Outcome
Long term side effects (cardiovascular disease, osteoporosis, fat redistribution, raised cholesterol, liver damage, and kidney damage, brain effects)	Long term effects	‘‘I wouldn’t want it to interfere with my organs, and that’s my concern about HIV meds or any long-term meds that I’m prescribed for an ongoing period of time [.] I would rather be on herbal drugs than to lose my kidneys and liver.’’[49] [Interviewer:] ‘‘Let’s assume [.] you would go to a regimen which means twenty-seven tablets, but there would be not such a high risk for having liver problems, and your renal system would be great, would you do it?’’ [Subject:] ‘‘I would try it, yes.’’ [49]	Long-term effect	Outcome
Willingness to accept higher pill burden in exchange for reduced long-term effect	The trade-off for reduced long term effect			
Restricted outdoor activities	Concerns related to drug side effects	‘‘If [.] this is a medicine that I have to take for a better quality of health and it interrupts my sleep for any period of time, it wouldn’t be acceptable. I couldn’t tolerate it.’’ [49] ‘‘Dizziness is a problem. I worry about falling.’’ [49] ‘‘You start to learn okay, [if] I know that the diarrhea is going to hit 2 h after I take my medcines, then I’m going to prepare for it.’’ [49] [Interviewer:] ‘‘So there’s a drug which would cause dizziness, but you could go down to one tablet in the morning, and there’s a drug which doesn’t cause dizziness, but you would have to take two tablets two times daily.’’ [Subject:] ‘‘Two tablets two times a day. I probably would go with the two tablets twice a day.’’ [49] [Interviewer:] ‘‘Let’s assume your drug actually results in sleeping problems. You go to your doctor [.]. He says, well, you know, there’s something else, but co-pays are real expensive. We’ve got to have some co-pay. $100.00?’’ [Subject:] ‘‘Yes.’’ [Interviewer:] ‘‘$150.00?’’ [Subject:] ‘‘Yes.’’ [Interviewer:] ‘‘$200.00? $250.00?’’ [Subject:] ‘‘My stopping point would be 200.’’ [49]	Side effect	Outcome
Restricted physical activity				
Selection of place and time of medication due to fainting				
Medication interfere with employed and non-employed work				
Stopped working due to side effects				
Drug interruption due to side effects				
Willingness to accept higher pill burden in exchange for reduced side effect	The trade-off for reduced side effect			
Willingness to pay for reduced side effects				
Skip doses while in the town, drink, or take illicit drugs	Social life inconvenience	‘‘One respondent, for example, reported being unable to take his scheduled dose because he was stuck in traffic and another reported that she had missed a dose because she had been unable to take a break from her job as a cashier.’’ [45] ‘‘You’re supposed to take that with a meal [. But] I never eat dinner at the same time. It’s just; it’s kind of an inconvenience.’’ [49] ‘‘Many noted taking medications with more complex regimens were problematic when they had to take medications with meals.’’ [45] ‘‘One subject reported that she had stopped taking her medications because she felt so nauseous and weak that she was unable to care for her newborn baby (she subsequently switched to a regimen with milder side-effects).’’ [45] ‘‘One said he felt embarrassed to take his medications in front of his kids and their friends.’’ [45]	Inconvenience	Structure and outcome
Drug interference with family and social life				
Skipping doses due to important engagements				
Food requirement	Food inconvenience			
Missing drug due to inconvenient time	Time inconvenience			
Drug stopping due to child care priority	Child care inconvenience			
Forget to take the drug due to child-caring				
Drug–drug interaction	Pharmacologic concern	‘‘My major concern is the interaction between other medicines because I have other conditions. I take other medicines. And they’re very strong, and I think that in the long-run, I can have a lot of complications.’’ [42] ‘‘Participants reported drug interactions in their medical history and highlighted the importance to not only check for interactions with HIV medicines, but also for medicines for comorbidities.’’ [49]	Drug interaction	Outcome
Coformulation of ART with chronic disease drugs	ARVs also treating other chronic conditions	“Several patients expressed a desire to co-formulate their ART with medication for another chronic illness like high blood pressure: I am wondering since high blood pressure is prevalent in the African American community, if they can have an antiviral medication that also helps with high blood pressure.” [42] “The other thing is I would like for or want this medicine to be in a convenient 3, 6, or 12-months injections.” [42]	Novel ART delivery approach	Structure and outcome
Hypertension controlling ART drug				
Patients want injectable drugs at 3,6 or 12 months	Injectable drugs reduce the frequency of visit			

#### Structure aspect of antiretroviral therapy

*Source of information on ART* One study highlighted the source of information on ART could affect the preferences of PLHIV regarding the ART service provision [39].

*Preferred place for ART service* Two studies evaluated a preferred place for ART service. Participants preferred health facility-based service to home-based ART service since this model gives the patients the opportunities to have hospital education and preparation before initiating a drug, meet and interact with colleagues, discuss with providers, have access to psychosocial support, and avoid stigma and discrimination [37, 39].

*Preferred person to deliver ART service* One study asked participants whom they preferred to deliver ART service. Participants’ choice of the service provider (health worker, trained PLHIV or foreigner) varied depending on the issues related to knowledge on ART, training, experience, encouraging patients to feel free to go to health facilities and avoiding a feeling of shyness, and maintaining confidentiality and interaction with patients [39].

*Cost of ART medications* Three studies evaluated the costs related to ART services [39, 42, 49]. Most patients preferred either a reduced or free drug cost. Some patients, however, were willing to pay whatever amount required as the drug is available.

*Time spent at ART clinics and times (hours and days) of operation* Two studies evaluated the waiting and clinic operation times at health facilities [37, 39]. Participants preferred shorter waiting times to obtain ART and flexible clinic hours.

*Visit frequency* One study asked participants about their preferred frequency of visits for ART pick-ups. Participants chose less frequent appointments (once or twice a year visit with larger supplies of ART dispensed at each visit) [37].

*Pill burden and pill size* One study evaluated the trade-off participants have on the pill size and pill burden. Some participants had a concern about swallowing big pills and most preferred single tablets. Some of them were willing to accept a higher pill burden in exchange for reduced side or long-term effects [49].

*Drug administration* Three studies examined the preference for drug administration [35, 42, 49]. Some participants preferred the drug in the form of a liquid, capsule, or injection to avoid swallowing of pills, lower the intake frequency and/or avoid a feeling of burden due to daily drug-taking and unpleasant drug taste.

*ART packaging* One study asked participants regarding their preference and recommendations for ART packaging [35, 37]. Participants identified three attributes of ART packaging that increased anticipated HIV stigma and prompted self-repackaging, including visual identification, bulkiness, and the rattling noise produced by ART pill bottles.

#### Process aspect of antiretroviral therapy

*Provider’s attitude* Two studies examined the providers’ attitude towards PLHIV while delivering care. Participants needed a nice approach and respectful care and maintained confidentiality, and being requested to bring a family member of their own choice [37, 39].

*Participants/others seen at the same visit* One study evaluated the preference for individualized versus group-based ART models. Participants preferred individualized ART models to group-based models due to privacy concerns [37].

*Patient involvement, relationships with providers, and shared decision making* One study examined the preference for patient involvement, relationships with their providers, and practice for shared decision making. Participants preferred good relationships and open communication with their providers [49].

#### Outcome aspect of antiretroviral therapy

*Efficacy* Three studies evaluated the efficacy of ARVs [42, 45, 49]. Participants needed their medication to control the HIV virus.

*Side and or long-term effects* One study examined both the side effects and long-term effects of taking the ART drugs [49]. Participants were concerned with the side effects of drugs. They preferred drugs with reduced or no side effects. They were willing to accept and or pay for reduced side effects [49]. Patients were also concerned with long-term effects and willing to accept and or pay for reduced long-term health effects [49].

*Drug–drug interaction* Two studies evaluated the participants’ preference towards drug–drug interaction between ARVs or ARVs with other medications. Participants have a strong concern about drug–drug interactions [42, 49].

Besides, the attributes of inconvenience and novel ART delivery methods were clustered into structure and outcome dimensions of ART service delivery.

*Inconvenience* Two studies asked the participants’ preference regarding convenience while taking medications. Participants mentioned their concern about inconvenience related to social life, food requirement, time in taking drugs, and child care activity [45, 49].

*Novel ART delivery methods* One study highlighted the importance of novel ART delivery approaches. Participants needed novel delivery of ART services, including coformulation of ART with chronic diseases drugs and injectable drug options [42].

### Integration of quantitative evidence and qualitative evidence

**Fig. 2:**
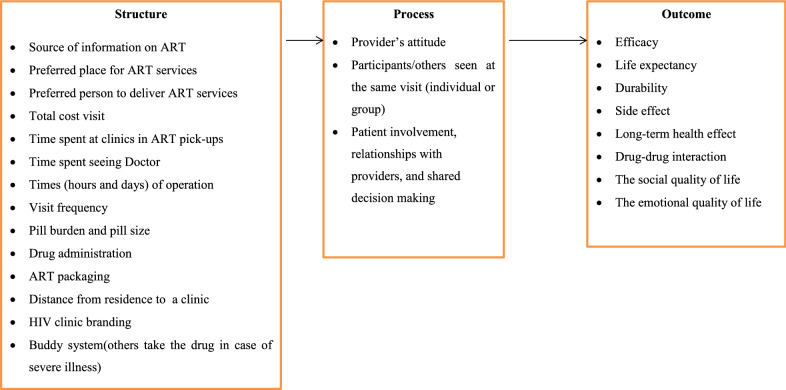
Donabedian model for ART Service Provision

The findings from individual quantitative and qualitative syntheses complement each other. Regarding the classification of attributes, the outcome aspect of ART took two-third of the share in the quantitative synthesis, whereas the structure aspect of ART took half of the share in the qualitative synthesis. The qualitative evidence explained well why the patients prefer or did not prefer a certain aspect of antiretroviral therapy service provision across the included quantitative studies. Attributes such as HIV clinic branding, accessibility to the clinic, time spent in seeing the doctor, and buddy system from the quantitative evidence were not explored in the qualitative studies and could therefore be investigated in future qualitative studies. On the other hand, the source of information on ART, packaging of ART and self-repackaging, and patient involvement, relationships with providers, and shared decision-making themes of the qualitative evidence were not tested in the quantitative studies. These factors would have implications for future discrete choice experiments. Figure 2 summarizes the attributes derived from both qualitative and quantitative evidence using a Donabedian framework.

## Discussion

Overall, this systematic mixed studies review identified several attributes underlying antiretroviral therapy choice in PLHIV. The conceptual attributes used by previous studies were clustered into the structure, process, and outcome domains of ART service delivery. The type of attribute and its relative importance on patients’ preferences varied across the included studies, which may have several reasons, such as the method of analysis, the selection procedure of the attributes and their levels, and/or the specific research question of the study.

### Structure attributes

In this review, health facility-based models of ART were highly valued than community-based models. It is consistent with previous evidence [16, 56]. This type of valuation could continue overstretching the existing health system and might create a barrier in scaling up ART to where PLHIV live and work hence deter achievement of 95-95-95 targets by 2030. Respondents’ choice of service provider type was influenced by the providers’ characteristics which are in line with previous evidence [57]. Healthcare workers were more preferred for their knowledge of drugs and disease, whereas trained PLHIV and foreigners (outside of their community) were preferred for their breaking down barriers and maintaining confidentiality. This highlights a difference in patient priority and has implications for patient-centered care. Regarding the source of information for ART, the majority of the participants in this review received information from health care providers, whereas some of them got information from TV and Radio. This finding is in line with a previous nationwide study where the majority of the participants ranked doctors in their top three information sources, HIV positive counselors and magazines next, and brochures and newsletters as last [58].

An increased total cost of visits (transportation, direct medical costs, and costs of childcare) was negatively associated with respondents’ choice of ART service, which is comparable with the previous systematic review [59]. This has implications for access to service and insurance coverage and further progress towards universal health coverage. Furthermore, less frequent clinic visit was highly preferred by respondents and is comparable with earlier reviews [16, 60]. The WHO’s differentiated service delivery initiative has also recognized the positive impacts of appointment spacing ART delivery models on patient and health system efficiencies [10]. Similarly, shorter travel distance was found to be preferred by participants, which is comparable with the previous review [16]. This has implications for access to service and demand for increased investment in community-based ART distribution models.

Regarding the waiting time to access antiretroviral drugs and clinical consultations, participants had more value on reduced waiting time, as similarly reported in previous reviews [14, 16]. This has implications for patient satisfaction. Similarly, respondents chose flexible or convenient clinic hours, including extra hours and weekends, which is consistent with an earlier systematic review [14]. This has policy implications on the health workforce and other resource allocation to increase service availability beyond the standard routine practice hours and days. Furthermore, participants preferred the availability of a buddy system (others take the drug in case of severe illness). From a policy perspective, this suggests that involving family members in care to maintain continuity of care as needed might improve patient drug adherence hence good treatment outcomes. HIV clinic branding was negatively associated with patient preference for ART service provision. This has implications for stigma reduction and care optimization since clinic branding might be a barrier to ART service utilization and adherence.

Reduction in pill burden was valued highly by patients. However, this would not continue as a concern since the current medication is based on fixed-dose single-tablet combinations. Besides, a smaller pill size was preferred by respondents. This implies for future patient-centered pharmaceutical drug formulation to ease medication swallowing. Patients also preferred injectable or liquid forms of drugs to reduce pill burden, avoid swallowing pills and unpleasant taste or reduce intake frequency. This has implications for drug innovators to bring new ARV options. In this review, patients had less preference on current ART packaging due to privacy issues and prioritized practicing self-repackaging. However, this could have a negative effect on the patient outcomes as a previous study reported an association of patient-initiated repackaging of ART with virological failure and ART drug resistance [61]. A patient-friendly pharmaceutical pack design is needed in future drug development investments, as inferred from this review.

### Process attributes

Good providers’ attitude was found positively associated with patients’ choice for ART service. This is consistent with the previous reviews [14, 16]. This has important implications for bringing interventions to continue enhancing providers’ empathy and positivity. Similarly, patients valued more their involvement and making a shared decision in HIV treatment and having good relationships with their providers, which is in line with the WHO’s people-centered health care policy framework [62] and a previous review [14]. This inferred that the shared decision model is appropriate in complex ART decisions. In this review, PLHIV were willing to accept individualized models than group-based models to reduce HIV stigma and discrimination even though group-based models were initially designed for reducing patients’ waiting time while receiving care. It is in line with a systematic review undertaken in sub-Saharan Africa [59]. This highlights much effort is needed to scale up group-based ART initiatives to enhance better patient outcomes.

### Outcome attributes

In this systematic review, patients highly valued effective ARV drugs, which is in line with a previous systematic review [63]. Also, the long duration of the drugs was highly valued. Likewise, increased quality and quantity of life were valued more considered as the important attributes underlying HIV drugs. Beyond the potency, increased life expectancy, and quality of life benefits of ARVs, patients also preferred the drugs to be with no or reduced side effects, long-term health problems, and drug–drug interactions. This is in agreement with the previous systematic review [63]. The WHO’s consolidated guidelines on the use of antiretroviral drugs for treating and preventing HIV infection [5] also acknowledged the above-mentioned attributes of HIV medications. This has implications for accommodating patient preferences in future drug discovery and development efforts by balancing the benefits and harms of treatment options.

Besides, this review found that novel ART delivery methods and inconvenience while taking medications as the relevant attributes affecting the preference of PLHIV on ART service delivery. A study conducted in the United States and Canada supports our review that patients preferred the long-acting injectable treatment regimen to avoid daily taking of drugs or reminders of having HIV [64]. Similarly, a systematic review found that HIV treatment fatigue occurred due to inconvenient scheduling, adverse side effects, and lifestyle changes which might affect patients’ choice of ART service delivery [65].

### Strengths and limitations of the review

This mixed-methods systematic review incorporated studies using both qualitative and quantitative methodologies to get a comprehensive understanding of the aspects of ART service delivery considered important by PLHIV in previous studies. This has the advantage of generating more robust implications for practice, research, and policymaking. This review has two noticeable limitations. First, as with the limitations of any systematic review, there is the possibility of incomplete retrieval of identified research due to the scope of the search terms and the databases searched. Second, there might be a probability of selection bias as only published studies in the English language were included.

## Conclusions

This review gives an overview of patients’ preferences for ART service provision features. Patients on ART had different values on the structure, process, and outcome components of antiretroviral therapy. The relative importance of each attribute used in the previous studies, as well as the patients’ preferences for ART service delivery characteristics, varied across the included studies. Thus, policymakers and practitioners should be aware of the aspects of ART that are considered as particularly important by the patients and the trade-offs, they are willing to make between various aspects of ART. Moreover, this review can be helpful for researchers planning to undertake a DCE in ART service since it gives a comprehensive picture of ART service delivery attributes and levels.

## Supplementary Information

**Additional file 1.** Search strategy for PubMed, Embase, Web of Science, and CINAHL databases.

**Additional file 2.** Data extraction tool.

**Additional file 3.** Mixed Methods Appraisal Tool (MMAT) checklist.

## Data Availability

All data generated or analyzed during this study are included in this published article and its additional files.

## Abbreviations

AIDS: Acquired Immunodeficiency Syndrome
ART: Antiretroviral therapy
ARV: Antiretroviral
DCE: Discrete choice experiment
HIV: Human Immunodeficiency Virus
PLHIV: People living with HIV
WHO: World Health Organization
