# Supplementary material for: Patients’ preferences for antiretroviral therapy service provision: a systematic review
Source: Cost Eff Resour Alloc. 2021 Aug 30;19:56. doi: 10.1186/s12962-021-00310-7 (PMC8404280; doi:10.1186/s12962-021-00310-7)
Supplement: Supplementary file 1 — Additional file 1. Search strategy for PubMed, Embase, Web of Science, and CINAHL databases. [file 12962_2021_310_MOESM1_ESM.docx]

**Additional File 1: Search Strategies**

**PubMed**

| # | **Searches** | **Results** |
| --- | --- | --- |
| 1 | Patient* | 182,953 |
| 2 | (''antiretroviral therapy'' OR ''antiretroviral therapy''[MeSH Terms] OR ''antiretroviral treatment'' OR ''human immunodeficiency virus therapy'' OR ''HIV treatment'' OR ''HIV medication'' OR ''HIV/AIDS therapy'' OR ''HIV/AIDS treatment'' OR ''differentiated antiretroviral therapy'') | 222,165 |
| 3 | (prefer* OR ''patient preference'' OR ''stated preference'' OR ''stated choice' | 12,826 |
| 4 | (quantitative OR qualitative OR ''mixed- methods'' OR ''discrete choice experiment'' OR ''ranking study'' OR ''swing weighting study'' OR ''analytical hierarchy process'' OR ''best-worst scaling'' OR ''adaptive conjoint analysis'') | 28,219 |
| 5 | #1 AND #2 AND #3 AND #4 | 423 |

***CINAHL***

| **#** | **Searches** | **Results** |
| --- | --- | --- |
| **S1** | "patient*" | 1,008,999 |
| **S2** | "''antiretroviral therapy*''" | 13,461 |
| **S3** | "''antiretroviral treatment''" | 1,657 |
| **S4** | "''human immunodeficiency virus therapy''" | 2 |
| **S5** | "''HIV treatment''" | 1,416 |
| **S6** | "''HIV medication''" | 5,977 |
| **S7** | "''HIV/AIDS therapy''" | 2 |
| **S8** | "''HIV/AIDS treatment''" | 116 |
| **S9** | "''differentiated antiretroviral therapy''" | 4 |
| **S10** | S2 OR S3 OR S4 OR S5 OR S6 OR S7 OR S8 OR S9 | 16,718 |
| **S11** | "prefer*" | 59,040 |
| **S12** | "''patient prefer*''" | 3,093 |
| **S13** | "''stated preference''" | 147 |
| **S14** | "''stated choice''" | 17 |
| **S15** | S11 OR S12 OR S13 OR S14 | 59,044 |
| **S16** | "quantitative" | 74,849 |
| **S17** | "qualitative" | 138,862 |
| **S18** | "''mixed- methods''" | 10,956 |
| **S19** | "''discrete choice experiment''" | 604 |
| **S20** | "''ranking study''" | 7 |
| **S21** | "swing weighting" | 3 |
| **S22** | "''analytical hierarchy process''" | 33 |
| **S23** | "''best-worst scaling''" | 102 |
| **S24** | "''adaptive conjoint analysis''" | 22 |
| **S25** | S16 OR S17 OR S18 OR S19 OR S20 OR S21 OR S22 OR S23 OR S24 | 195,751 |
| **S26** | S1 AND S10 AND S15 AND S25 | 51 |

***Embase***

| **#** | **Searches** | **Results** |
| --- | --- | --- |
| **1** | 'patient*' | 10,927,473 |
| **2** | 'highly active antiretroviral therapy' | 40,498 |
| **3** | antiretroviral AND treatment | 57,087 |
| **4** | human AND immunodeficiency AND virus AND therapy | 206,391 |
| **5** | hiv AND treatment | 133,067 |
| **6** | hiv AND medication | 11,975 |
| **7** | hiv AND aids AND therapy | 74,389 |
| **8** | hiv AND aids AND treatment | 54,177 |
| **9** | differentiated AND antiretroviral AND therapy | 492 |
| **10** | #2 OR #3 OR #4 OR #5 OR #6 OR #7 OR #8 OR #9 | 265,125 |
| **11** | prefer* | 615,906 |
| **12** | patient AND prefer* | 158,330 |
| **13** | stated AND preference | 2,607 |
| **14** | stated AND choice | 3,497 |
| **15** | #11 OR #12 OR #13 OR #14 | 618,099 |
| **16** | quantitative | 1,058,424 |
| **17** | qualitative | 348,728 |
| **18** | mixed- AND methods | 204,533 |
| **19** | discrete AND choice AND experiment | 3,016 |
| **20** | ranking AND study | 21,030 |
| **21** | swing AND weighting AND study | 41 |
| **22** | analytical AND hierarchy AND process | 558 |
| **23** | 'best worst' AND scaling | 324 |
| **24** | adaptive AND conjoint AND analysis | 124 |
| **25** | #16 OR #17 OR #18 OR #19 OR #20 OR #21 OR #22 OR #23 OR #24 | 1,486,274 |
| **26** | #1 AND #10 AND #15 AND #25 | 468 |
| **27** | #26 AND ('article'/it OR 'article in press'/it) | 311 |

***Web of Science***

| **#** | **Searches** | **Results** |
| --- | --- | --- |
| 1 | TS=patient* | 6,473,486 |
| 2 | TS= ''antiretroviral therapy*'' | 68,969 |
| 3 | TS= ''antiretroviral treatment'' | 37,571 |
| 4 | TS=''human immunodeficiency virus therapy'' | 38,111 |
| 5 | TS=''HIV treatment'' | 81,971 |
| 6 | TS=''HIV medication'' | 10,085 |
| 7 | TS=''HIV/AIDS therapy'' | 10,049 |
| 8 | TS=''HIV/AIDS treatment'' | 10,282 |
| 9 | TS=''differentiated antiretroviral therapy'' | 597 |
| 10 | #9 OR #8 OR #7 OR #6 OR #5 OR #4 OR #3 OR #2 | 133,712 |
| 11 | TS= prefer* | 913,816 |
| 12 | TS= ''patient prefer*'' | 123,307 |
| 13 | TS= ''stated preference'' | 47,012 |
| 14 | TS= ''stated choice'' | 76,504 |
| 15 | #14 OR #13 OR #12 OR #11 | 979,241 |
| 16 | TS=quantitative | 1,082,209 |
| 17 | TS= qualitative | 515,647 |
| 18 | TS=''mixed- methods'' | 367,619 |
| 19 | TS=''discrete choice experiment'' | 4,986 |
| 20 | TS=''ranking study'' | 167,319 |
| 21 | TS=''swing weighting study'' | 879 |
| 22 | TS=''analytical hierarchy process'' | 6,888 |
| 23 | TS=''best-worst scaling'' | 685 |
| 24 | TS=''adaptive conjoint analysis'' | 247 |
| 25 | #24 OR #23 OR #22 OR #21 OR #20 OR #19 OR #18 OR #17 OR #16 | 1,935,179 |
| 26 | #25 AND #15 AND #10 AND #1 | 208 |
| 27 | #25 AND #15 AND #10 AND #1 | 186 |
